# Infusion of hESC derived Immunity‐and‐matrix regulatory cells improves cognitive ability in early‐stage AD mice

**DOI:** 10.1111/cpr.13085

**Published:** 2021-07-07

**Authors:** Jing Liu, Zongren Hou, Jun Wu, Kailun Liu, Da Li, Tingting Gao, Wenjing Liu, Bin An, Yun Sun, Fan Mo, Liu Wang, Yukai Wang, Jie Hao, Baoyang Hu

**Affiliations:** ^1^ State Key Laboratory of Stem Cell and Reproductive Biology Institute of Zoology Chinese Academy of Sciences Beijing China; ^2^ Institute for Stem Cell and Regeneration Chinese Academy of Sciences Beijing China; ^3^ University of Chinese Academy of Sciences Beijing China; ^4^ Beijing Institute for Stem Cell and Regenerative Medicine Beijing China; ^5^ Savaid Medical School University of Chinese Academy of Sciences Beijing China; ^6^ National Stem Cell Resource Center Chinese Academy of Sciences Beijing China

**Keywords:** AD early stage, Alzheimer's disease, immunity‐and‐matrix regulatory cells, mesenchymal stem cell, stem cell therapy

## Abstract

**Objectives:**

In this study, we administered immunity‐and‐matrix regulatory cells (IMRCs) *via* tail vein (IV) and intracerebroventricular (ICV) injection to 3‐month‐old 5×FAD transgenic mice to assess the effects of IMRC transplantation on the behaviour and pathology of early‐stage Alzheimer's disease (AD).

**Materials and methods:**

Clinical‐grade human embryonic stem cell (hESC)‐derived IMRCs were produced under good manufacturing practice (GMP) conditions. Three‐month‐old 5×FAD mice were administered IMRCs *via* IV and ICV injection. After 3 months, the mice were subjected to behavioural tests and electrophysiological analysis to evaluate their cognitive function, memory ability and synaptic plasticity. The effect of IMRCs on amyloid‐beta (Aβ)‐related pathology was detected by thioflavin‐S staining and Western blot. Quantitative real‐time PCR, ELISA and immunostaining were used to confirm that IMRCs inhibit neuroinflammation. RNA‐seq analysis was performed to measure changes in gene expression and perform a pathway analysis in response to IMRC treatment.

**Results:**

IMRC administration via tail vein injection significantly ameliorated cognitive deficits in early‐stage AD (5×FAD) mice. However, no significant change was observed in the characteristic pathology of AD in the ICV group. Plaque analysis revealed that IMRCs did not influence either plaque deposition or BACE1 expression. In addition, IMRCs inhibited inflammatory responses and reduced microglial activation in vivo.

**Conclusions:**

We have shown that peripheral administration of IMRCs can ameliorate AD pathology and associated cognitive deficits.

## Graphical abstract

Clinical‐grade human embryonic stem cell (hESC)‐derived immunity‐and matrix‐regulatory cells (IMRCs) alleviate cognitive symptoms of early‐stage AD mice by anti‐inflammatory effects in the peripheral system.

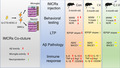

## 1 INTRODUCTION

Alzheimer's disease (AD) is the most commonly diagnosed age‐related neurodegenerative disease and is characterized by progressive memory decline and cognitive dysfunction. Amyloid deposits, neurofibrillary tangles comprising hyperphosphorylated tau protein and excessive inflammatory response are the main pathologic hallmarks of AD.^1^ To date, there is no curative therapy for this disorder. All the drugs currently used to treat AD only ameliorate the clinical symptoms and have no preventive effect on its pathology.^2^, ^3^, ^4^, ^5^, ^6^ These observations underline the need to identify new therapeutic targets for the treatment of AD.

Stem cells have emerged as a potential therapy for a range of neurological insults; however, their application in AD remains limited and the mechanisms underlying the cognitive benefits of stem cells remain to be elucidated.^7^, ^8^ Recent studies have highlighted the immune regulatory potential of mesenchymal stem cells (MSCs).^9^, ^10^, ^11^ MSCs have emerged as promising agents in combating AD.^12^, ^13^, ^14^, ^15^, ^16^ MSCs are adult stem cells that can differentiate into several mesenchymal cell lineages and have the capacity for self‐renewal. At present, the main categories of stem cells that can be used in research include bone marrow mesenchymal stem cells (BM‐MSCs),^17^ adipose‐derived mesenchymal stem cells (AD‐MSCs),^18^ and human umbilical cord mesenchymal stem cells (hUC‐MSCs). We have previously identified a hESC‐derived MSC‐like population with unique abilities in modulating immunity and regulating extracellular matrix production, which we named immunity‐and‐matrix regulatory cells (IMRCs).^19^ We showed that the intravenous delivery of IMRCs inhibits both pulmonary inflammation and fibrosis in mouse models of lung injury in a dose‐dependent manner. Additionally, IMRCs were superior to both primary hUC‐MSCs and the FDA‐approved drug pirfenidone in treating lung injury, and displayed an excellent efficacy and safety profile in both mice and monkeys. Given the public health crises involving pneumonia, acute lung injury and acute respiratory distress syndrome, our findings suggested that IMRCs are ready for clinical trials to assess their efficacy and safety in the treatment of lung disorders. This study was performed to assess whether IMRCs may also be a suitable therapeutic candidate for AD treatment. We showed that IMRCs administered to AD mice *via* tail vein injections reduced neuronal loss and improved memory capacity and cognitive deficits by suppressing inflammation.

## 2 MATERIALS AND METHODS

### 2.1 Animals

Male 5 × FAD mice (MMRRC ID 034848‐JAX‐008730) in a C57BL/6 background were obtained from The Jackson Laboratory. These mice overexpress mutant human amyloid‐beta (A4) precursor protein (APP) and human presenilin‐1 (APPSwFlLon, PSEN1*M146L*L286V). Only male mice were used because of the gender‐specific differences in the progression of AD pathology. Mice were housed at the Laboratory Animal Center of the Institute of Zoology under standard conditions, including a 12:12‐hour light/dark cycle, and were allowed free access to food and water. All animal experiments were approved by the Animal Care and Use Committees of the Institute of Zoology, Chinese Academy of Sciences.

### 2.2 Cell culture

SH‐SY5Y and BV2 cells were maintained in DMEM supplemented with 10%‐15% foetal bovine serum (FBS) and 100 units penicillin and 100 µg/mL streptomycin and cultured at 37℃ with 5% CO_2_.

### 2.3 Co‐culture assay

BV2 cells were placed on the insert of a Transwell plate (0.4‐μm polycarbonate filter, Corning, MA, USA), while the IMRCs were placed on the lower chamber. The medium was additionally treated with or without LPS (500 ng/mL) for 12 h during BV2 co‐culture with IMRCs. At the end of the experiment, BV2 cells were washed with PBS and mRNA expression was detected by qPCR.

### 2.4 Preparation of oligomeric Aβ

Oligomeric Aβ_1–42_ (oAβ_1–42_) was prepared as previously described.^20^ Pure Aβ_1–42_ peptides were dissolved in hexafluoroisopropanol and volatilized to form a peptide membrane, followed by dissolution in 20 µL of DMSO. Ice‐cold phenol‐free Ham's F‐12 cell culture medium was then added, and the sample was incubated at 4℃ for 24 hours to obtain a 1 mmol/L oAβ_1–42_ stock solution.

### 2.5 Cell viability assay

Cell viability was measured by 3‐(4,5‐dimethylthiazol 2‐yl)‐2,5‐(diphenyltetrazolium bromide) (MTT) assay. SH‐SY5Y cells were seeded into 96‐well plates at 2 × 10^4^ cells/well and then treated with 10 μmol/L oAβ_1–42_ with or without conditional medium (CM). At the end of the incubation period, MTT solution (final concentration: 0.5 mg/mL) was added to each well following which the cells were incubated for 4 hours at 37℃. After removing the medium, DMSO was added to dissolve the blue formazan product. Absorbance was measured with a microplate reader at 490 nm. Cell survival rates were expressed as the percentage of the absorbance of treated cells to that of control cells.

### 2.6 Transplantation of IMRCs into 5×FAD mice

IMRC suspensions or NaCl alone were administered to 5×FAD mice at the age of 3 months. For stereotactic brain injection (n = 8), mice were anaesthetized with isoflurane and fixed on a stereotaxic apparatus (RWD Life Science, Shenzhen, China). Using a 10‐μL syringe (Hamilton) and an automated syringe pump (KD Scientific, MA, USA), 2 μL (approximately 2.5 × 10^5^ cells) of the IMRC suspension was injected at a rate of 0.2 μL/min bilaterally into the ventricle using the following stereotaxic coordinates: 0.4 mm posterior to the bregma, 1.0 mm bilateral to the midline and 3 mm ventral to the skull surface. The syringe was kept in place for 5 min after the injection. For intravenous injection (n = 9 for both IMRCs and control injections), 5×FAD mice were injected with a total of 5 × 10^6^ IMRCs suspended in 200 μL of 0.9% NaCl or 0.9% NaCl alone through the lateral tail vein. Behavioural tests and electrophysiological tests were carried out 90 days after cell transplantation.

### 2.7 Morris water maze test

The water maze was a circular pool (120 cm in diameter, 60 cm in height) with a white inner surface. The escape platform (10 × 10 cm) was fixed in the centre of one quadrant and submerged 1 cm below the water surface. In training sessions, mice were allowed to navigate in the tank to find the hidden platform. If a mouse failed to find the platform within 60 s, it was gently guided to the platform and allowed to stay there for 25 s. Each mouse performed eight training trials per day, starting from different quadrants, for 5 days. Test sessions were performed 24 h after the last training trial. The test session was a single probe test in which the platform was removed and mice were allowed to swim in the tank for 60 s. Behaviours were analysed by video tracking software (EthoVision, Noldus, Netherlands). Latency to find the platform during the training trials and the time spent in each quadrant during the test session were recorded.

### 2.8 Y‐maze test

The Y‐maze apparatus consisted of three radial 30‐cm‐long arms (named as starting, novel and other arms) originating from the central space to form a ‘Y’ shape. Mice were placed into the starting arm to explore the maze based on the rodent's innate curiosity to explore novel areas. Briefly, mice were placed into the starting arm to explore and allowed 5 minutes to freely locate the novel arms using spatial clues (training period). After a 2‐hour interval, mice were placed into the Y‐maze again as part of the training period protocol to evaluate spatial memory. Time, distance, enter times and movement tracks were recorded by an automated video tracking system.

### 2.9 Novel object recognition

Novel object recognition is widely used in rodents to measure short‐term memory and learning, the preference for novelty and the influence of the hippocampus in the process of recognition.^21^ The test was performed in a square‐shaped open‐field box with objects located opposite the starting point. Briefly, mice were allowed to explore two identical objects (cylinders) in the open field for 10 minutes (learning period). After a 24‐hour interval, mice were allowed to explore one familiar object (cylinder) and one novel object (cuboid) as part of the learning period protocol. The time spent exploring familiar and novel objects and the movement tracks of the mice were recorded using a tracking system.

### 2.10 Open‐field activity test

The open‐field test measures the exploration of a new environment and anxious behaviour and is based on the idea that mice naturally prefer to be near a protective wall rather than exposed to danger out in an open field. The test was performed in a square‐shaped open‐field box (as described in the previous section) comprising an inside square (150 mm × 150 mm) as the ‘centre area’ and an outside square as the ‘surrounding area’. Each mouse was gently placed on the floor and allowed to freely explore the area for 10 minutes to investigate their spontaneous locomotor activity. Their overall time spent, distance travelled and movement tracks in the centre and surrounding areas were measured by a tracking system.

### 2.11 Electrophysiology

Long‐term potentiation (LTP). Hippocampal slices were prepared as previously described.^22^ Briefly, the brain, including the two hippocampi, was removed and placed into an ice‐cold bath containing artificial cerebrospinal fluid (ACSF) (234 mmol/L Sucrose, 2.5 mmol/L KCl, 1.25 mmol/L NaH_2_PO_4_•2H_2_O, 25 mmol/L NaHCO_3_, 25 mmol/L D‐glucose, 0.5 mmol/L CaCl_2_·2H_2_O and 10 mmol/L MgSO_4_) supplemented with 95% O_2_ and 5% CO_2_ (saturation pH: 7.2‐7.4). Then, the base of the tissue block was fixed in a vibratome holder containing ice‐cold ACSF, the blade's height was adjusted, and the hippocampus was transversely cut with a vibrating slicer (Leica, VT 1000 S, Wetzlar, Germany) at a thickness of 380 µM. When the slice to be the hippocampus, according to the experimental needs, slices of hippocampus specific area cut (eg, CA3‐CA1 region). The prepared brain sections were transferred to a 32℃ bath containing saturated recording ACSF (125 mmol/L NaCl, 2.5 mmol/L KCl, 1.25 mmol/L NaH_2_PO_4_·2H_2_O, 25 mmol/L NaHCO_3_, 10 mmol/L D‐glucose, 2 mmol/L CaCl_2_·2H_2_O and 1.5 mmol/L MgSO_4_) supplemented with 95% O_2_ and 5% CO_2_ (saturation pH: 7.2‐7.4) and incubated for 30 minutes. The brain sections were then placed at 22‐23℃ for at least 1.5 hours, after which the brain sections were moved one by one to the recording chamber and were perfused with saturated incubation fluid (ACSF). The temperature of the extracellular solution (ACSF) was maintained at 31 ± 1℃ and flowed to the recording chamber at the rate of 6 mL/min.

The brain sections were placed in a recording chamber where the temperature was controlled by a water bath heating system and the perfusate was flowing at the rate of 6 mL/min. The recording glass electrode was pulled by a 10‐cm‐long boron silicate glass capillary tube (GB 150F‐8P, Sutter instrument, CA, USA). The outer diameter of the glass tube was 1.5 mm and the inner diameter 1.1 mm. The inner wall of the glass tube had fibres attached to facilitate the filling of the electrode fluid.

### 2.12 Immunohistochemistry

Mice were anaesthetized with 2.5% avertin (200 mg/kg body weight) and then perfused with cold PBS followed by 4% paraformaldehyde (PFA). The brains were subsequently removed and post‐fixed in 4% PFA overnight and then dehydrated with 30% sucrose. Finally, the brains were coronally sectioned into 40‐μm‐thick slices using a cryostat (Leica SM2010 R) and stored at −20℃ in cryoprotective storage solution (125 mL of ethylene glycol, 125 mL of glycerol and 150 mL of 0.1 mol/L phosphate buffer) until use. For immunohistochemical staining, the sections were washed three times with PBS, blocked using 5% BSA and 1% Triton X‐100 in PBS for 2 hours at room temperature, and incubated with primary antibodies (in 1% BSA and 0.2% Triton X‐100 in PBS) overnight at 4℃. The primary antibodies used include rabbit polyclonal anti‐Iba1 (1:1,000; 019‐19741; Wako, Saitama, Japan), mouse monoclonal anti‐MOAB‐2 (1:1,000; ab126649; Abcam, Cambridge, UK) and rabbit monoclonal anti‐vGlut1 (1:500; ab180188; Abcam, Cambridge, UK). After washing, the sections were incubated with secondary antibodies conjugated to Alexa Fluor 488/568/594/647 in blocking solution containing DAPI (D1306; Invitrogen, CA, USA).

### 2.13 Western blotting

Cultured cells or brain tissues were homogenized in ice‐cold RIPA Lysis and Extraction Buffer (89901; Thermo, CA, USA) supplemented with a protease inhibitor cocktail (78439; Thermo, CA, USA). The protein concentration was determined using a BCA assay kit (23250; Thermos, CA, USA). Protein samples were separated by 8%–12% SDS–PAGE, blotted onto a polyvinylidene fluoride membrane (Millipore), blocked for 60 minutes in 5% milk and incubated overnight at 4℃ with rabbit monoclonal anti‐MOAB‐2 (1:2,000; ab126649; Abcam), mouse monoclonal anti‐β‐actin (1:4,000; A5441; Sigma), rabbit monoclonal anti‐BACE1 (1:1,000; ab183612; Abcam) and mouse monoclonal anti‐GAPDH (1:4,000; AF0006; Beyotime) antibodies. After three washes with TBST, the membranes were incubated with horseradish peroxidase‐conjugated goat anti‐mouse or goat anti‐rabbit secondary antibodies at room temperature for 2 hours. The immunoreactive bands were detected using an enhanced chemiluminescence reagent (ECL, Pierce) and quantified using ImageJ software.

### 2.14 Thioflavin‐S staining

For the detection of Aβ plaques, brain sections were first incubated with 0.1% thioflavin‐S (Thio‐S, Sigma) in the dark for 5 minutes in 50% ethanol, followed by two washes with 50% ethanol and three washes with PBS, and then subjected to antibody staining as described above.

### 2.15 RNA isolation and RT‐qPCR

Total RNA was isolated from mouse brain using the RNAprep Pure Kit (Qiagen, Mannheim, Germany) following the manufacturer's instructions. To remove residual DNA contamination, 1 mg of total RNA was treated with 50 units of DNase I (Yeasen, China) at 37℃ for 30 minutes. The purified RNA was reverse‐transcribed into cDNA using a Revert Aid First Strand cDNA Synthesis Kit (Yeasen). qPCR was performed with a SYBR Green Real‐time PCR Master Mix (Yeasen) in a StepOne Plus Real‐Time PCR System (Applied Biosystems) using the following cycling conditions: 94℃ for 2 minutes, followed by 40 cycles of 94℃ for 5 s, 56℃ for 15 s and 72℃ for 20 s. Fluorescence data were acquired at the 72℃ step and during the melting curve programme. GAPDH and beta‐actin served as the reference genes. Triplicate PCRs were performed for each of three independently purified RNA samples. Quantitative PCR primers were designed to amplify fragments of approximately 100‐200 bp (Table 1) using Primer‐BLAST online software.

**TABLE 1:** Primers used for RT‐qPCR

Gene	Sequence (5′‐3′)
Mouse *IL‐6* F 141	CCCCAATTTCCAATGCTCTCC
Mouse *IL‐6* R 141	CGCACTAGGTTTGCCGAGTA
Mouse *IL‐1β* F 138	TGCCACCTTTTGACAGTGATG
Mouse *IL‐1β* R 138	TGATGTGCTGCTGCGAGATT
Mouse *TNF‐α* F 151	CGAGTGACAAGCCTGTAGCC
Mouse *TNF‐α* R 151	ACAAGGTACAACCCATCGGC
Mouse *GAPDH* F 123	AGGTCGGTGTGAACGGATTTG
Mouse *GAPDH* R 123	TGTAGACCATGTAGTTGAGGTCA
Mouse *β‐actin* 281	ACAGTCCGCCTAGAAGCAC
Mouse *β‐actin* 281	CGTTGACATCCGTAAAGACC
Mouse *Arg1* F 185	CTCCAAGCCAAAGTCCTTAGAG
Mouse *Arg1* R 185	AGGAGCTGTCATTAGGGACATC
Mouse *IL‐10 F* 105	GCTCTTACTGACTGGCATGAG
Mouse *IL‐10 R* 105	CGCAGCTCTAGGAGCATGTG

### 2.16 RNA‐seq analysis

RNA and library preparation, clustering, sequencing and data analyses were performed by the BGI Experimental Department. Sequencing libraries were generated using the NEBNext UltraTM RNA Library Prep Kit for Illumina (NEB) according to the manufacturer's protocol, and index codes were added to attribute sequences to each sample. After cluster generation, the prepared libraries were sequenced on an Illumina platform, and 125 bp/150 bp paired‐end reads were generated. A differential expression analysis between the two groups was performed using the DESeq2 package in R (1.16.1). Genes with an adjusted *P*‐value of <.05 (obtained by DESeq2) were considered to be differentially expressed. A corrected *P*‐value of .05 and an absolute fold change in 2 were set as the thresholds for significantly differential expression. The KEGG is a database resource for understanding the high‐level functions and utilities of a biological system. Data were analysed using the clusterProfiler package in R to test the enrichment of DEGs in KEGG pathways. A gene ontology (GO) enrichment analysis was implemented with the same package.

## 3 RESULTS

### 3.1 hESC‐derived IMRCs rescued Aβ‐induced neural cell damage and LPS‐induced inflammation *in* *vitro*

**FIGURE 1:**
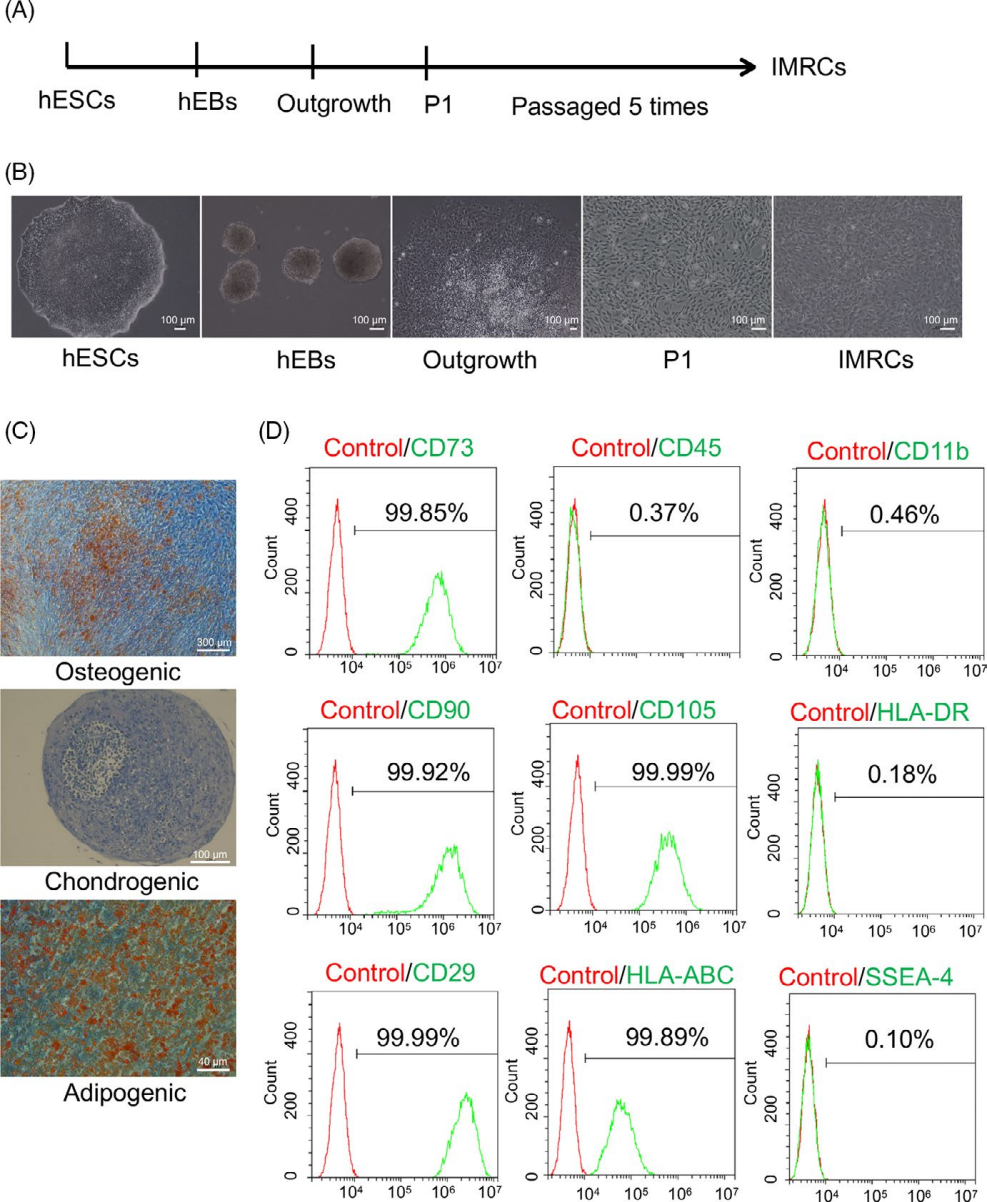
Derivation of immunity‐and‐matrix regulatory cells (IMRCs) from human embryonic stem cells (hESCs). A, Different stages of the IMRC derivation protocol. B, Changes in cell morphology during the induction of hESCs into IMRCs. C, The osteogenic, chondrogenic and adipogenic differentiation potential of the IMRC lines generated. D, Flow cytometric analysis of marker expression in IMRCs (passage 5). IMRCs are CD11b−/CD45−/HLA–DR−/CD90+/CD29+/CD73+/CD105+ cells. hEBs, human embryoid bodies. P1, passage 1

**FIGURE 2:**
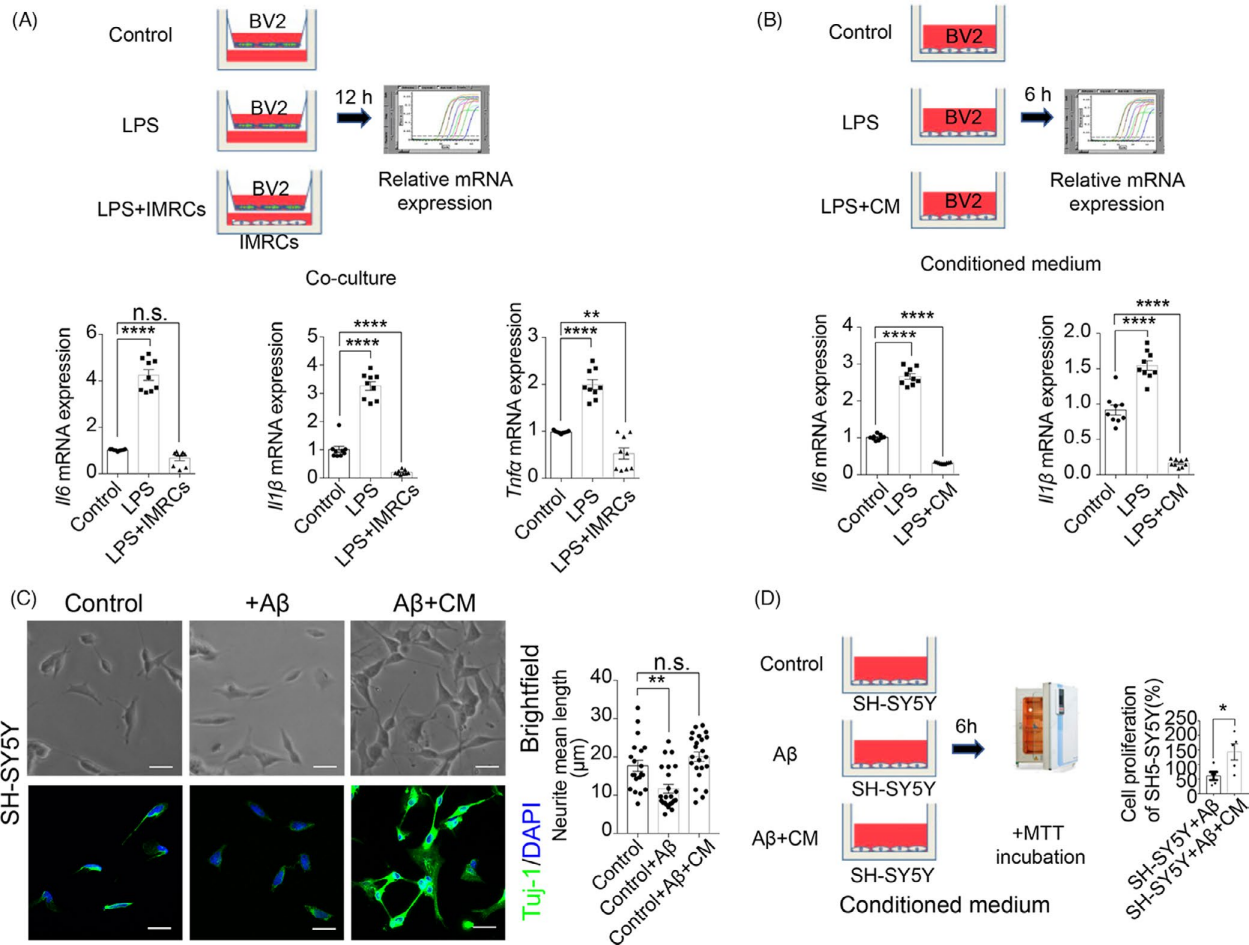
Immunity‐and‐matrix regulatory cells (IMRCs) could rescue amyloid‐beta (Aβ)‐induced neural cell damage and lipopolysaccharide (LPS)‐induced inflammation in vitro. (A), IMRCs inhibited LPS‐induced inflammation in a co‐culture model. Schematic of a BV2 and IMRCs co‐culture model and the quantification of mRNA expression. n = 9 repeats, means ±SEM, one‐way ANOVA with Tukey's multiple comparison test; n.s., nonsignificant, **p <.01, ****P <.0001. (B), Conditional medium (CM) of IMRCs reduces LPS‐induced inflammation in BV2 microglial cells. Schematic of the experimental workflow (upper) and the quantification of mRNA expression (lower). n = 9 repeats, means ± SEM, one‐way ANOVA with Tukey's multiple comparison test, ****p <.0001. (C), Representative brightfield (upper) and fluorescence (lower) images of the morphology of SH‐SY5Y cells. The quantification of the neurite mean length is shown on the right. n = 22 cells per group; data are shown as means ±SEM. All comparisons were made by one‐way ANOVA with Tukey's multiple comparison test; n.s., nonsignificant, **p <.01. (D), CM of IMRCs reduced Aβ‐induced neural cell damage in vitro. Schematic of the MTT assay (left) and the quantification of cytotoxicity by MTT assay (right). n = 5 repeats, means ±SEM, *p <.05; unpaired Student's *t* test

Studies have indicated that potential associations exist between MSCs and IMRCs. Several of these have reported that MSCs inhibit LPS‐induced pulmonary or inflammatory immune responses in AD.^23^, ^24^, ^25^ In this study, hESC‐derived IMRCs were prepared as described in our previous research (Figure 1A–D).^19^ The clinical hESC line (Q‐CTS‐hESC‐2) was prepared as previously described.^26^ To assess whether IMRCs could inhibit inflammation and the neurotoxic effects of Aβ, we co‐cultured IMRCs and BV2 cells (immortalized microglia) in Transwell inserts and determined the anti‐inflammatory potential of IMRCs in the presence of LPS. The mRNA levels of proinflammatory factors (*Il‐6*, *Il1β*, *Tnf*) were markedly reduced in BV2 cells treated with IMRCs and LPS when compared with those treated with LPS alone, indicating that IMRCs could rescue LPS‐induced inflammation in vitro (Figure 2A). The same effect was observed with CM treatment (Figure 2B), which indicated that the positive influence of IMRCs on inflammation was due to cell secretion. To assess Aβ‐induced neural cell damage, SH‐SY5Y cells were treated with CM containing 20 μmol/L oAβ_1–42_.^27^ At the start of treatment (0 hour), SH‐SY5Y cells exhibited polygonal morphology, with long axons and abundant dendrites. After 12 hours of oAβ_1–42_ treatment, cell bodies had adopted a round morphology with shortened, bent, and fractured axons. In contrast, the cells in the CM treatment group displayed and maintained a morphology similar to that of controls (Figure 2C). In addition, MTT assay results confirmed the increased viability of SH‐SY5Y cells after CM treatment (Figure 2D). Together, these findings suggested that IMRCs have significant anti‐inflammatory and neuroprotective properties.

### 3.2 IMRCs improved spatial learning and memory ability in early‐stage AD mice

**FIGURE 3:**
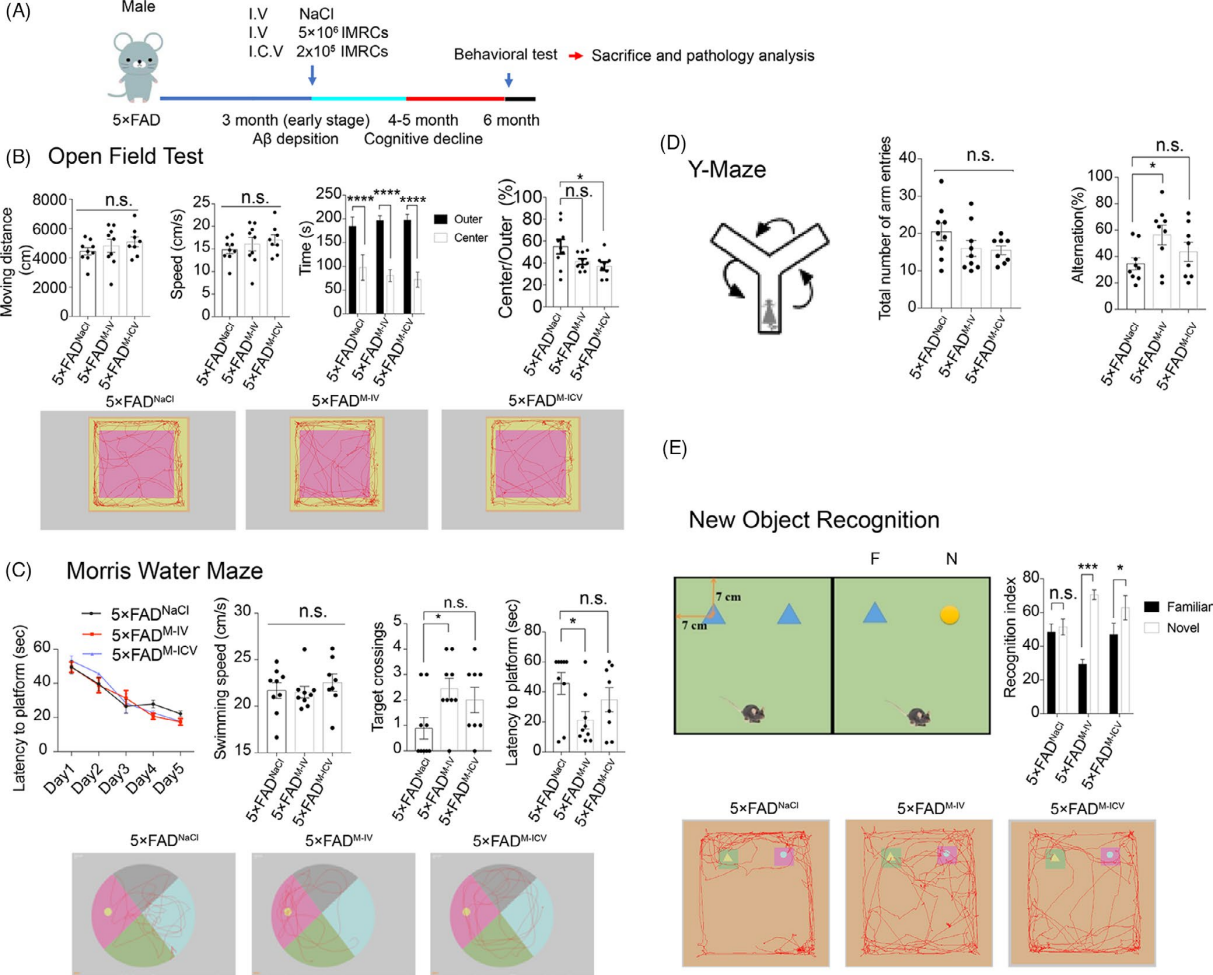
Immunity‐and‐matrix regulatory cell (IMRC) administration *via* tail vein injection improved spatial learning and memory ability in 5×FAD mice at the early stages of Alzheimer's disease. (A), Schematic illustrating the chronological order for intravenous (IV) and intracerebroventricular (ICV) IMRC administration or IV NaCl treatment (5×FAD^M‐IV^, 5×FAD^M‐ICV^ and 5×FAD^NaCl^ groups, respectively); and Morris water maze (MWM), Y‐maze, open‐field and object‐recognition testing. (B), Results of the open‐field test for the three groups showing that there were no differences in moving speed among the groups. (C), Results of the Morris water maze test for the three groups. The typical route of escape (lower), mean daily escape latencies (upper left) and platform crossings and latency times (upper right) are shown. There was no difference in the average swimming speed among the three groups (upper left 2). (D), Schematic of the Y‐maze (left), the number of arm entries (middle) and the percentage of alternations [calculated as (actual alternations/maximum alternations‐2) × 100] (right). E, Schematic of the new object recognition (NOR) test (upper left), per cent time exploring familiar and novel objects in the NOR test (upper right) and a representative movement track (lower). In each graph. n = 8‐9 mice per group; all data are shown as means ± SEM. All comparisons were made by one‐way ANOVA with Tukey's multiple comparison test; n.s., nonsignificant, *p <* *.05; **p <* *.01; ***p <* *.001

Next, we investigated whether IMRC injection could alleviate cognitive deficits in the early stages of AD. 5×FAD mice, widely used in preclinical research, exhibit amyloid deposition in the brain and behavioural deficits at 1 and 4 months of age, respectively.^28^ Here, we used ≤3‐month‐old 5×FAD mice as a model of early‐stage AD (early stage of AD pathology).^29^, ^30^ IMRCs were administered *via* tail vein (5 × 10^6^ cells/mouse) and intracerebroventricular (2.5 × 10^5^ cells/mouse) injection. As a control, NaCl was injected *via* the tail vein. Because 5×FAD mice gradually develop memory deficits that correlate with Aβ deposition at 6 months of age, behavioural tests were performed 90 days after injection when the animals were 6 months old (Figure 3A). In the open‐field test, no significant differences were found for the time spent in the centre square, moving speed or moving distance among the three groups, indicating that IMRCs did not ameliorate motor abilities, exploratory behaviour or anxiety in 5×FAD mice (Figure 3B). In the Morris water maze test,^31^ during the training phase (4 trials per day for 5 successive days), both the IV and ICV groups showed improved latency to the platform compared with that of control mice. There was no significant difference in swimming speed between the 3 groups of mice. In the subsequent probe test phase, control and ICV groups displayed a higher latency to targets and fewer target crossings compared with their littermates in the IV group (Figure 3C). To assess short‐term memory, Y‐maze tests were performed. Mice in the IV group also showed improved performance in the Y‐maze (Figure 3D). The novel objective recognition test reflects the learning and memory ability of mice based on their natural tendency to explore novel objects instead of familiar ones when exposed to a novel environment. When administered *via* tail vein injection, IMRCs ameliorated memory deficits in the AD mice as evidenced by the results of the novel object recognition test (Figure 3E). Together, these findings suggested that, compared with the NaCl (control) and ICV groups, 5×FAD mice administrated IMRCs by tail vein injection displayed significantly improved spatial learning and memory abilities.

### 3.3 IMRCs enhanced hippocampal synaptic plasticity in 5×FAD mice

**FIGURE 4:**
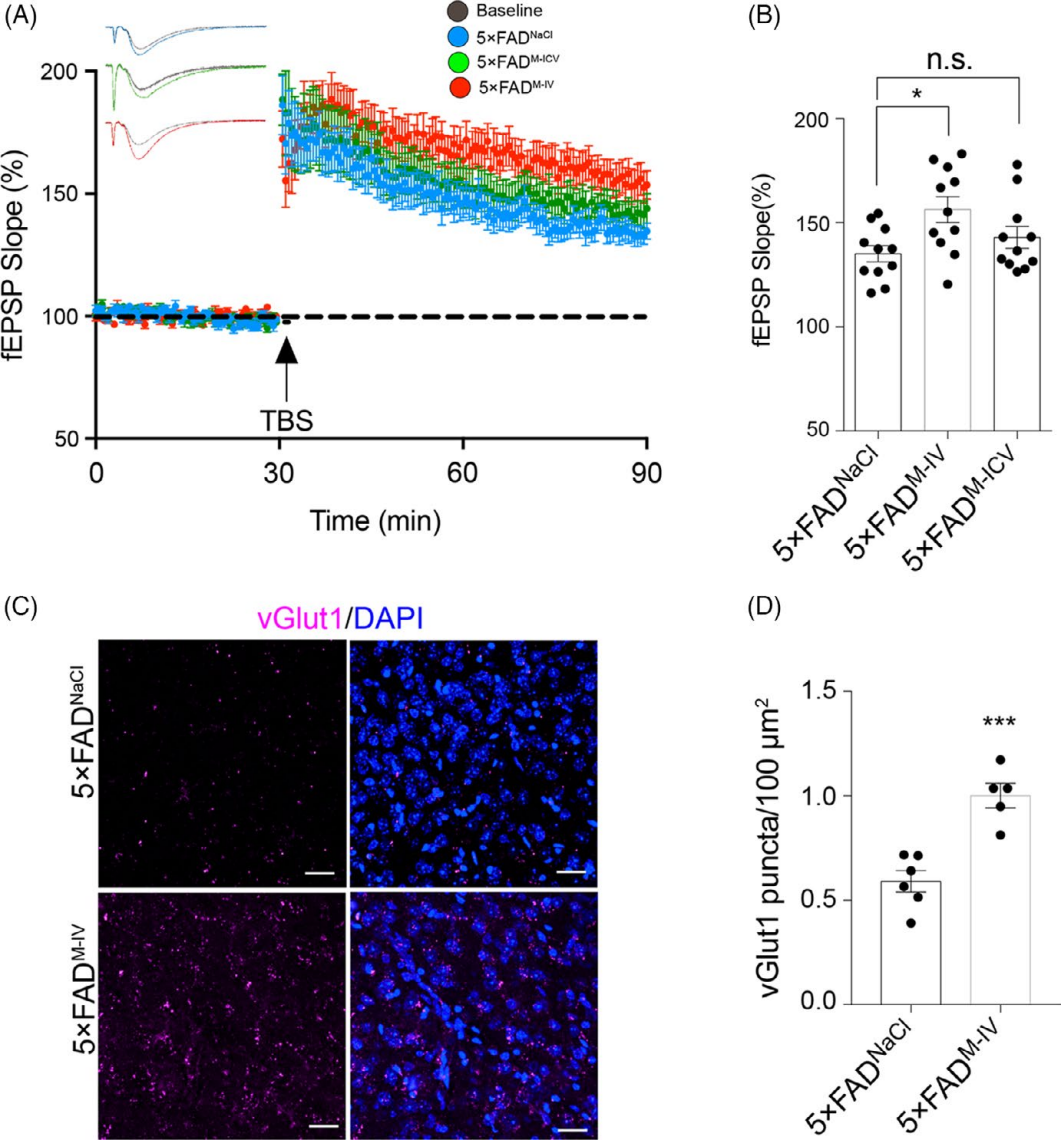
Immunity‐and‐matrix regulatory cells (IMRCs) enhanced hippocampal synaptic plasticity in 5×FAD mice. (a), (b), Long‐term potentiation (LTP) in the hippocampal CA1 region was induced by high‐frequency stimulation (HFS); 5×FAD^M‐IV^, intravenous (IV) IMRC administration; 5×FAD^M‐ICV^, intracerebroventricular (ICV) IMRC administration; 5×FAD^NaCl^, IV NaCl treatment. (A), Averaged slopes of baseline normalized field excitatory postsynaptic potentials (fEPSPs). (B), Quantification of mean fEPSP slopes during the last 10 min of the recording after LTP induction (n = 10 slices per group from 3 mice, mean ± SEM, *p <* *.05; unpaired Student's *t* test). (C), (D), Representative confocal images of vGlut1immunostaining in the hippocampus of 5× FAD mice administered IMRCs (5×FAD^M‐IV^) or NaCl (controls; 5×FAD^NaCl^) by tail vein injection (c) and relative quantification (d). Pink, vGlut1; blue, DAPI (n = 5‐6 slices per group from 3 mice). Scale bar, 20 µm; data are presented as means ± SEM; ***P <* *.001, unpaired Student's *t* test

Long‐term potentiation (LTP) is characterized by a persistent increase in synaptic strength, a form of synaptic plasticity needed in learning and memory.^32^ At the neurophysiological level, AD mice consistently show impaired hippocampal LTP.^33^, ^34^ Consequently, we next asked whether IMRCs could also potentially improve synaptic plasticity in AD mice. To this end, we performed brain slice electrophysiology experiments. The NaCl (control) group exhibited a lower slope of evoked field excitatory postsynaptic potential (fEPSP) responses than the IV and ICV groups (Figure 4A, B). LTP quickly reached baseline levels in the NaCl group, but was maintained above baseline in the IV and ICV groups throughout the recording period. These results revealed that synaptic plasticity in AD mice was significantly enhanced when IMRCs was administered *via* tail vein injection. This conclusion was further confirmed by immunofluorescence staining, which showed an increased number of vGLUT1 puncta in hippocampal neurons of mice in the IV group compared with that in the other two groups (Figure 4C, D).

Combined, the behavioural and electrophysiological studies indicated that the administration of IMRCs *via* tail vein injection led to a significant improvement in spatial learning and memory compared with that in the control and ICV groups.

### 3.4 IMRCs did not affect Aβ pathology in 5×FAD mice

Aβ reflects the key neuropathological hall markers of AD pathology.^35^ Because BACE1 initiates the formation of Aβ,^36^, ^37^, ^38^ BACE1 inhibition is highly effective in reducing Aβ production. Aβ levels have been reported to be decreased following MSC‐based therapy.^16^, ^24^, ^25^ To investigate whether IMRC injection can affect BACE1 activity and, consequently, Aβ deposition, we examined BACE1 activity by immunoblot analysis.

**FIGURE 5:**
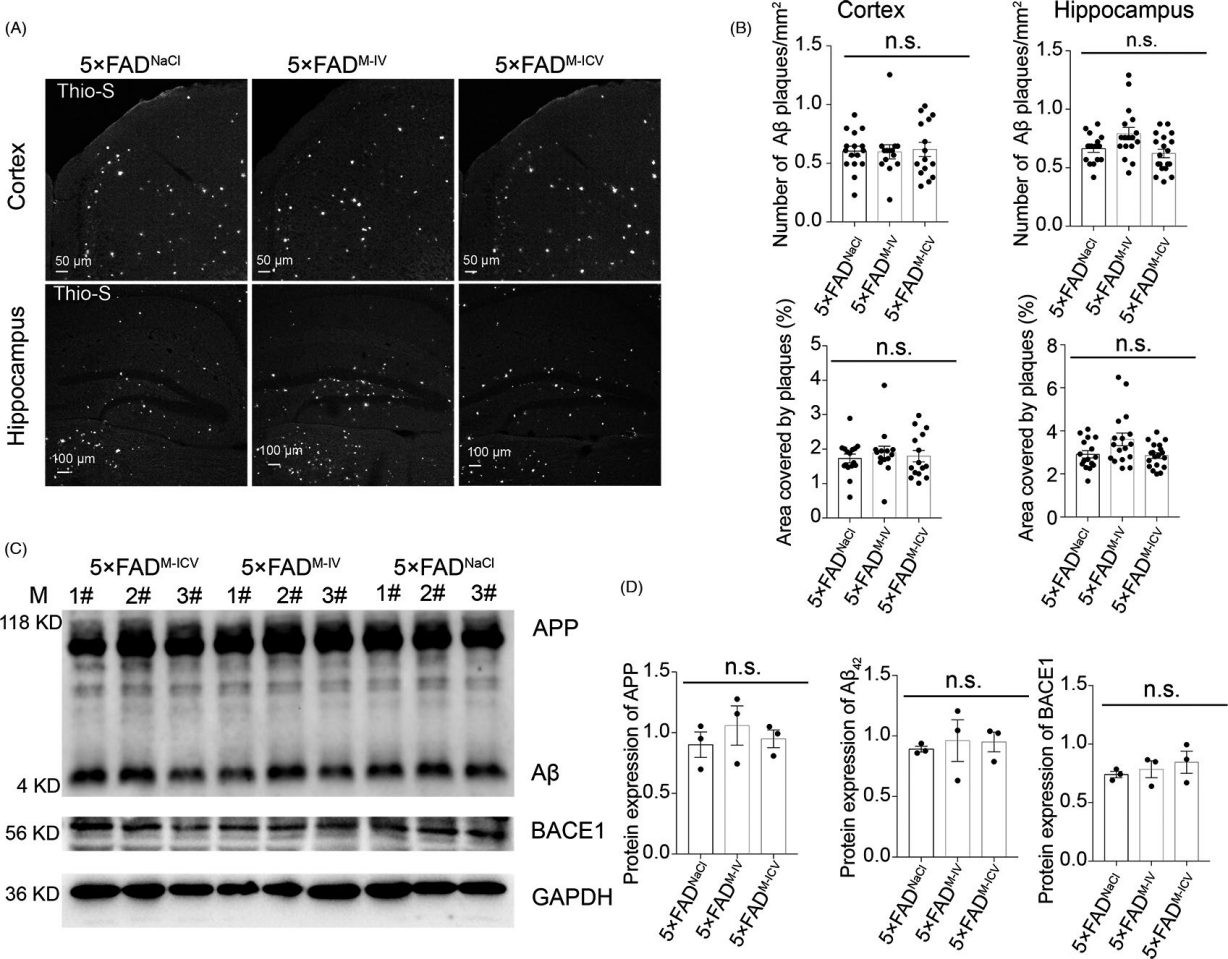
Immunity‐and‐matrix regulatory cell (IMRC) treatment does not alter the development of amyloid‐beta (Aβ)‐pathology in 5×FAD mice. (A), (B), Representative images of thioflavin‐S staining (A) and quantification (B) of the numbers and areas of Aβ plaques in the cortex and hippocampus; 5×FAD^M‐IV^, intravenous (IV) IMRC administration; 5×FAD^M‐ICV^, intracerebroventricular (ICV) IMRC administration; 5×FAD^NaCl^, IV NaCl treatment. n = 16 to 19 slices from 3 mice per group; data represent means ± SEM; one‐way ANOVA with Tukey's multiple comparison test; n.s., nonsignificant. Scale bars: 50 μm (upper); 100 μm (lower). (C), (D), Representative Western blots (C) and relative quantification (D) of Aβ expression levels in cortical tissues from each group. There was no significant reduction in Aβ deposition in 5**×**FAD mice (n = 3 mice per group; data represent means ± SEM; one‐way ANOVA with Tukey's correction; n.s., nonsignificant)

No significant reduction in Aβ deposition was seen among the three groups of AD mice at 6 months of age (Figure 5A, C). To investigate whether the IMRCs affected the amyloid load, we performed thioflavin‐S staining and observed no changes in the amyloid load either in the cortex or in the hippocampus (Figure 5A, B). Western blot results of BACE1 expression also showed no significant differences among the three groups of mice (Figure 5C, D). These results suggested that IMRC injection is likely to modulate AD pathology through a mechanism other than Aβ deposition. Notably, BACE1 inhibitors may pose a safety risk as, in addition to APP, BACE1 also mediates the cleavage of several other substrates that are important for normal physiology,^39^ indicating that IMRCs that do not affect BACE1 may be more suitable for clinical applications.

### 3.5 IMRCs decrease microglial activation in 5×FAD mice by suppressing inflammation

**FIGURE 6:**
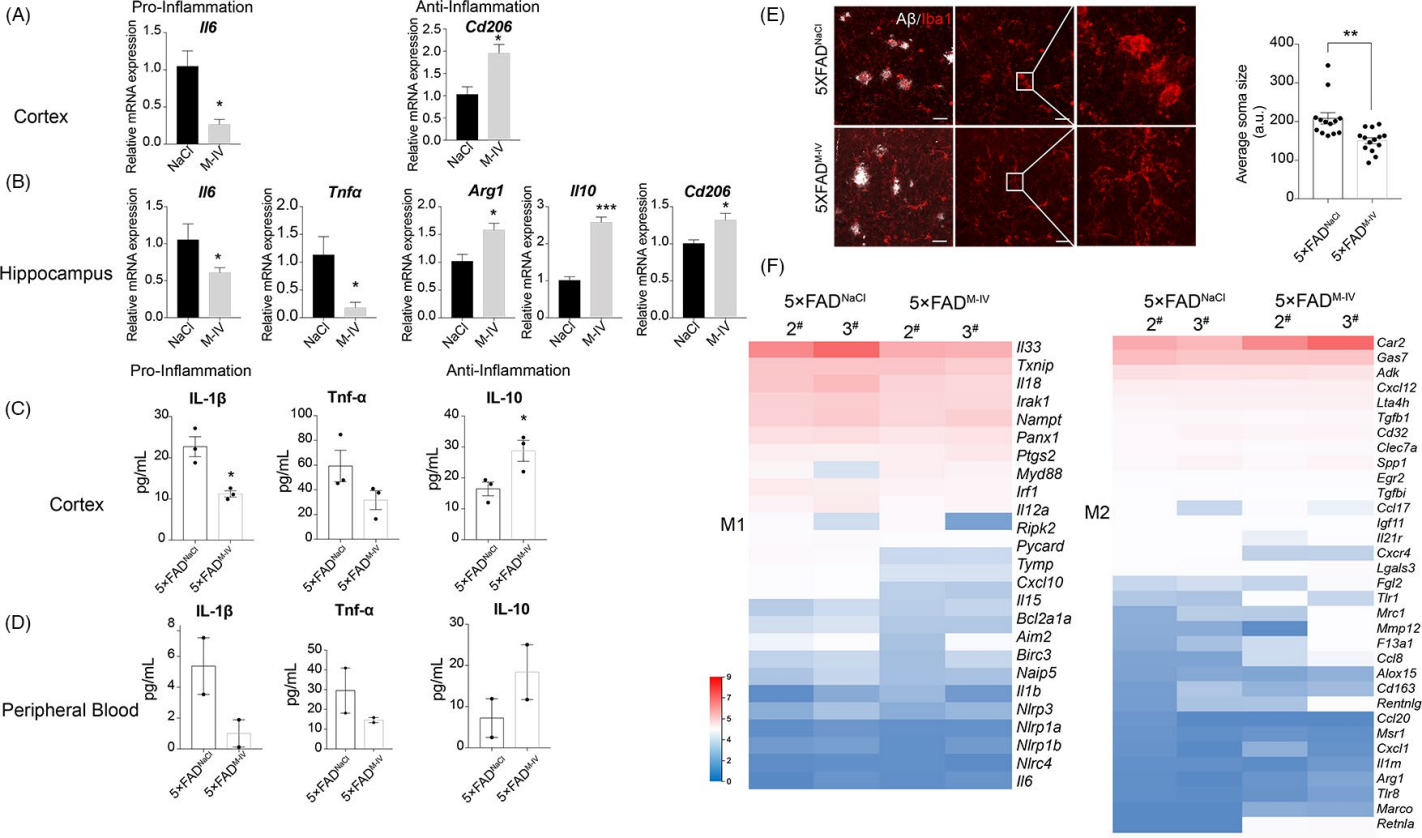
Immunity‐and‐matrix regulatory cells (IMRCs) can suppress inflammatory responses and decrease microglial activation in 5×FAD mice. (A), (B), Relative mRNA expression levels of the proinflammatory‐related genes *Il‐6* and *Tnfa* and the anti‐inflammatory‐related genes *Cd206*, *Il10* and *Arg1* in the cortex (A) and hippocampus (B); 5×FAD^M‐IV^, intravenous (IV) IMRC administration; 5×FAD^NaCl^, IV NaCl treatment. n = 3 mice per group; means ± SEM, *p < .05; unpaired Student's *t* test. (C), (D), ELISA analysis of proinflammatory and anti‐inflammatory cytokines in the cortex (C) and peripheral blood (D) after intravenous IMRC injection. n = 3 mice per group (cortex) or n = 2 mice per group (peripheral blood); mean ± SEM, *P <.05; unpaired Student's *t* test. E, Representative image of microglial cells in the brain of 5×FAD mice after IMRC treatment. Scale bars = 20 μm. IBA1 (red). Histograms comparing the reduction (shown as arbitrary units [a.u.]) in microglial cell body size in 5×FAD mice after IMRC treatment. n = 13‐14 slices per group from 3 mice; mean ± SEM, **P < .01; unpaired Student's *t* test. (f) The expression profile of key genes related to inflammation. Heatmap showing the expression of M1‐like and M2‐like markers in the brains of 5×FAD mice administered IMRCs (5×FAD^M‐IV^) or NaCl (5×FAD^NaCl^) *via* tail vein injection. n = 2 mice per group. All expression data are presented as log2 fold changes compared with control samples

Substantial evidence supports that Aβ pathology is a key factor in the progression of AD; however, the relationship between Aβ and AD remains contentious.^40^ The high failure rate for Aβ‐focused drug candidates for the treatment of AD indicates that Aβ may not be the optimal therapeutic target to combat this disease.^41^ Dysregulation of the inflammatory system in ageing and AD can also affect brain function and facilitate cognitive impairment. Inflammation is especially important as it occurs in pathologically vulnerable regions of AD brains and can influence AD development.^1^, ^42^, ^43^, ^44^, ^45^ Immunoactivity and microglia are suspected to also play a role in the pathology of AD.^1^, ^44^, ^46^, ^47^, ^48^ The results of our in vitro experiments suggested that IMRCs could inhibit the microglia‐mediated inflammatory response. To further investigate the mechanism in vivo, we sought to identify inflammation‐related factors in the hippocampus and cortex by RT‐qPCR and ELISA. In the cortex of mice in the IV injection group, the expression of *IL‐6*, encoding a proinflammatory factor, was downregulated, whereas that of CD206, which codes for an anti‐inflammatory factor, was upregulated (Figure 6A). In the hippocampus, meanwhile, the expression of the proinflammatory factors IL‐6 and TNF‐α was downregulated, whereas that of the anti‐inflammatory factors Arg1, IL‐10 and CD206 was increased (Figure 6B). ELISA for IL‐1β, TNF‐α and IL‐10 levels further confirmed these results (Figure 6C). These data clearly indicate that IMRCs can modulate the inflammatory response. As impaired cognitive function has been linked to central and peripheral inflammation,^49^ we also measured cytokine levels in peripheral blood by ELISA and found that the levels of proinflammatory factors were also decreased after IMRC injection (Figure 6D). Given that the activation of microglia has been implicated in neuroinflammation during the development of AD, we evaluated microglial activity by immunofluorescence. Microglia in the NaCl (control) group showed a typical activated morphology with hypertrophied cell bodies, whereas microglia in the IV group displayed decreased soma size, suggesting that IMRC injection *via* the tail vein can inhibit microglial activation (Figure 6E). RNA‐seq analysis revealed that the expression of most of the M1‐related (proinflammatory) markers^50^ was downregulated in the IV group compared with that of the NaCl group. The expression of M2 (anti‐inflammation)‐associated genes was upregulated (Figure 6F).

Taken together, the results demonstrated that IMRC treatment downregulated the expression levels of inflammatory factors in both the brain and the peripheral blood.

### 3.6 RNA‐seq analysis identified biological processes associated with the immune response

**FIGURE 7:**
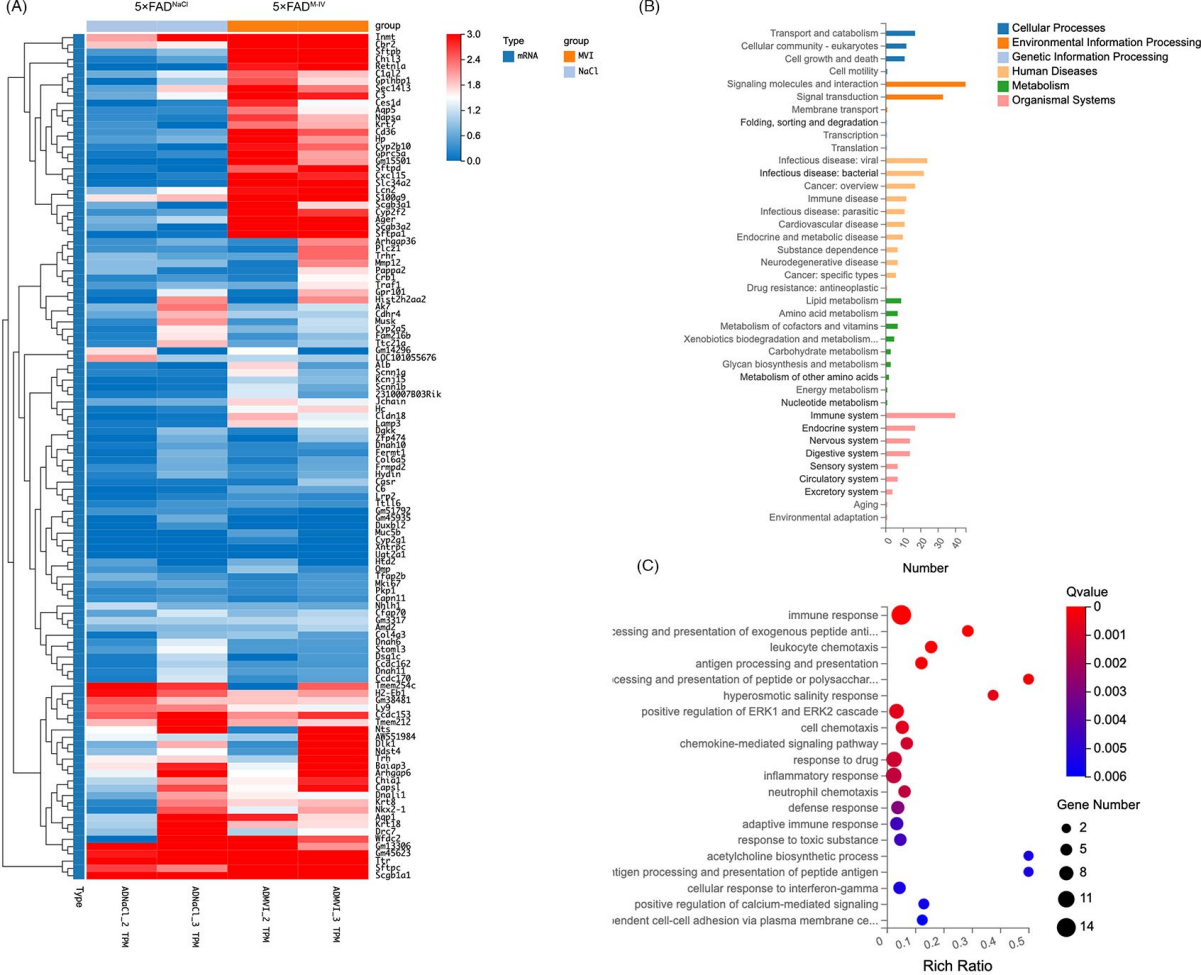
RNA‐seq analysis of 5×FAD mice after immunity‐and‐matrix regulatory cell (IMRC) treatment identified the biological process associated with the immune response. (A), The expression profile of two distinct clusters of differentially expressed genes (DEGs) between IMRC‐treated and control mice. Colouring indicates the log2‐transformed fold change. (B), KEGG pathway analysis evaluated for the representative profiles of genes involved in Cellular Processes, Environmental Information Processing, Genetic Information Processing, Human Diseases and Organismal Systems. (C), GO enrichment analysis in biological process (*q < 0.05; **q < 0.01)

To further investigate the role of IMRCs in AD pathology, we performed RNA‐seq assays on the brains of mice in the IV (n = 2) and NaCl groups (n = 2). Analysis of the generated heatmap showed that the two groups had distinct expression patterns (Figure 7A). After IMRC treatment, 134 genes were upregulated and 175 downregulated. Next, the differentially expressed genes (DEGs) were subjected to KEGG pathway and GO enrichment analysis (Figure 7B, C). The results showed that immune system‐associated genes were upregulated. Moreover, we observed an enrichment of pathways associated with neurodegenerative diseases, the immune system and the nervous system (Figure 7B). GO enrichment analysis of the DEGs revealed an abundance of genes associated with the biological processes of immune response, proinflammatory response, adaptive immunity response and interferon‐gamma response (Figure 7C). This conclusion is identical to the one drawn from our experimental data.

## 4 DISCUSSION

The Alzheimer's Association (AA) 2019 report projected that more than 100 million individuals worldwide will suffer from AD by 2050. Despite the heavy public disease burden associated with AD, there is no effective treatment for this disease. Over several decades, MSCs derived from umbilical cord, adipose tissue or bone marrow have shown potential for AD therapy.^18^, ^51^, ^52^, ^53^ Moreover, MSCs from different studies may have different ways of action, such as the reduction in neuro‐inflammation,^25^, ^54^ the elimination of Aβ,^25^ the promotion of autophagy‐associated and blood‐brain barrier recoveries,^55^ the upregulation of acetylcholine levels^56^ and the recovery of mitochondrial transport,^57^ indicating that these MSCs may possess disparate biofunctions. Moreover, MSC homing and migration ability are inconsistent in different studies.^58^, ^59^, ^60^ It has been reported that the homing of MSCs can be influenced by a variety of factors, such as the time and quantity of transplantation, the culture method, pre‐treatment and the transplantation method of MSCs.^61^, ^62^ These demonstrated that finding an alternative stable source of MSCs is crucial for the continued development of stem cell‐based therapies.

Different from adult tissue‐derived cells, MSC‐like cells differentiated from hPSCs have unique advantages in quality control and large‐scale production. We previously showed that hESC‐derived MSC‐like cells (IMRCs) exhibit stronger immunomodulatory effects in the treatment of lung injury and fibrosis compared with that of primary MSC populations.^19^ In this study, we report that IMRCs produced under GMP requirements can improve memory ability and enhance cognitive function in AD mice through the suppression of inflammation when administered at an early stage of the disease. Human ESCs provide a limitless supply of IMRCs through cell differentiation.

The efficacy of MSCs has been widely investigated in mouse models of late‐stage AD (from 7 to 19 months of age), and good results have been reported.^14^, ^15^, ^24^, ^25^, ^63^ In the past few years, substantial progress has been made to understand the pathophysiology and genetic basis of AD. The preclinical stage of AD is considered as the cellular phase.^64^ In this stage, alterations in neurons, microglia and astroglia drive the insidious progression of the disease before cognitive impairment is observed.^65^ The clinical trial suggests that it might be too late to treat AD and an effective treatment for AD might need an early intervention. Therefore, attempts must focus on increasing early‐stage diagnosis and treatment in AD.^66^, ^67^, ^68^ Herein, we showed that early intervention (3‐month‐old mice) can also play a role in disease prevention.

The therapeutic effects of IMRCs were evaluated through IV and ICV routes. All AD mice treated with peripheral MSC therapy exhibited progressive improvement in cognitive function. However, no significant change was observed in the characteristic pathology and symptoms of AD in the ICV group. These results indicated that the ameliorating effect of IMRCs may be related to their potent anti‐inflammatory effects in the periphery.

Besides therapeutic efficacy, safety is also an important therapy index.^69^, ^70^ IMRCs are superior to both primary UCMSCs and the FDA‐approved drug pirfenidone in treating lung injury and display an excellent efficacy and safety profile in both mice and monkeys. Regarding AD pathologies, IMRCs did not influence plaque deposition or BACE1 expression, which may be advantageous for AD treatment. Secretase inhibitors have mostly shown disappointing results in clinical trials with an observed worsening of cognitive functions and adverse drug reactions.^4^, ^71^, ^72^

In this study, we provided additional evidence for the potential of IMRCs as a novel candidate for use in cell‐based therapy. IMRCs have shown promise in the treatment of AD in vitro and in vivo. This is critical not only for new strategies aimed at AD prevention and early intervention but also for stem cell‐based treatment. Nevertheless, an extensive evaluation of safety, effectiveness and repeatability needs to be carried out before IMRCs can be widely applied in cytotherapy.

## CONFLICT OF INTEREST

The authors declare there is no competing interest, and all authors consented to publish the data.

## AUTHOR CONTRIBUTIONS

JL, ZH and JW collected and assembled the data, and wrote the manuscript; KL, TG, SY, FM and LW collected and assembled the data; WL provided cell sources; BA and DL contributed to IMRC transplantation and collected the data; YW, JH and BH: conceptualized and designed, analysed and interpreted the data, and gave final approval of the manuscript.

## Data Availability

The data that support the findings of this study are available from the corresponding author on reasonable request.
